# Real-World Outcomes and Prognostic Factors in Patients Receiving Nivolumab Therapy for Recurrent or Metastatic Head and Neck Carcinoma

**DOI:** 10.3390/cancers11091317

**Published:** 2019-09-06

**Authors:** Ryusuke Hori, Shogo Shinohara, Tsuyoshi Kojima, Hiroki Kagoshima, Morimasa Kitamura, Ichiro Tateya, Hisanobu Tamaki, Yohei Kumabe, Ryo Asato, Hiroyuki Harada, Yoshiharu Kitani, Takashi Tsujimura, Keigo Honda, Kazuyuki Ichimaru, Koichi Omori

**Affiliations:** 1 Department of Otolaryngology, Tenri Hospital, Nara 632-8552, Japan; 2 Department of Otolaryngology – Head & Neck Surgery, Kobe City Medical Center General Hospital, Hyogo 650-0047, Japan; 3 Department of Otolaryngology – Head & Neck Surgery, Graduate School of Medicine, Kyoto University, Kyoto 606-8501, Japan; 4 Department of Otolaryngology – Head & Neck Surgery, Kurashiki Central Hospital, Okayama 710-8602, Japan; 5 Department of Otolaryngology – Head & Neck Surgery, Hyogo Prefectural Amagasaki General Medical Center, Hyogo 660-8550, Japan; 6 Department of Otolaryngology – Head & Neck Surgery, National Hospital Organization Kyoto Medical Center, Kyoto 612-8555, Japan; 7 Department of Otolaryngology – Head & Neck Surgery, Medical Research Institute, Kitano Hospital, Osaka 530-8480, Japan; 8 Department of Otorhinolaryngology – Head & Neck surgery, Shizuoka General Hospital, Shizuoka 420-8527, Japan; 9 Department of Otolaryngology, Japanese Red Cross Otsu Hospital, Shiga 520-0046, Japan; 10 Department of Otolaryngology, Japanese Red Cross Wakayama Medical Center, Wakayama 640-8558, Japan; 11 Department of Otolaryngology – Head & Neck Surgery, Kokura Memorial Hospital, Fukuoka 802-8555, Japan

**Keywords:** Nivolumab, recurrent or metastatic head and neck carcinoma, squamous cell carcinoma, treatment-related adverse events, prognostic factor

## Abstract

Recently, a global phase III study demonstrated that nivolumab markedly improved patient outcomes in recurrent or metastatic head and neck carcinoma (RMHNC). However, the efficacy of nivolumab in patients who are ineligible for clinical trials is unknown. We investigated nivolumab efficacy in real-world patients and prognostic factors associated with the response to nivolumab. This study was conducted at 11 institutes associated with Kyoto University and its Affiliated Hospitals-Head and Neck Oncology Group. In total, 93 patients with RMHNC who received nivolumab between May 2017 and May 2018 were retrospectively reviewed. Objective response rate (ORR), overall survival, and progression-free survival (PFS) were evaluated. Univariate and multivariate analyses were performed to identify prognostic factors. The ORRs in patients with squamous cell carcinoma (SCC) and non-SCC were 21.8% and 0%, respectively. In patients with SCC and non-SCC, the 1-year PFS rates were 28.7% and 8.9%, respectively. The hazard ratio (HR) for risk of PFS events (SCC versus non-SCC) was 2.28 (95% confidence interval: 1.21–4.1; log-rank *p* = 0.007). Univariate and multivariate analyses revealed radiotherapy history, platinum-refractory carcinoma, and treatment-related adverse events (TRAEs) as important prognostic factors associated with PFS in patients with SCC. In a real-world setting, non-SCC and platinum-refractory carcinoma were associated with a poorer prognosis, and a history of radiotherapy to the primary tumor, and the occurrence of TRAEs were associated with a better prognosis. These findings could be useful for clinicians and patients when selecting a treatment strategy.

## 1. Introduction

Head and neck carcinoma (HNC) occur in various regions of the head and neck, including the lip, oral cavity, nasal cavity, paranasal sinuses, nasopharynx, oropharynx, hypopharynx, larynx, major salivary glands, and others. More than 600,000 patients are diagnosed with HNC annually worldwide [1]. In Japan, approximately 20,000 patients are affected by HNC annually [2]. Over 50% of patients who present with locoregionally advanced disease experience recurrence within three years [3,4,5]. In the Checkmate-141 clinical trial, nivolumab, an anti-programmed death-1 (PD-1) monoclonal antibody, yielded better results than existing chemotherapeutic agents [6]. Based on this trial, nivolumab was approved in March 2017 for the treatment of patients with recurrent or metastatic HNC (RMHNC) in Japan.

Checkmate-141 provided accurate and meaningful results and improved the outcomes of RMHNC treatment. However, more recently, clinical trials similar to Checkmate-141 have been conducted with more strict and complicated eligibility criteria [6,7,8]. The eligibility criteria in Checkmate-141 included recurrent or metastatic squamous cell carcinoma (SCC) of the head and neck of the oral cavity, pharynx, or larynx; platinum-refractory carcinoma; an Eastern Cooperative Oncology Group (ECOG) performance-status (PS) score of 0 or 1 [6]; this study populations represent only a minority of real-world patients. In fact, real-world patients included certain HNC subtypes, such as nasopharyngeal carcinoma, salivary gland carcinoma, and unknown primary carcinoma of the head and neck; non-SCC; non-platinum-refractory carcinoma; an ECOG PS score of 2 or 3. Therefore, the applicability of the clinical trial results to patients with RMHNC who do not precisely fit the eligibility criteria of pivotal clinical trials is unclear because the strict eligibility criteria in Checkmate-141 prevented enrollment of a broad patient population, even though real-world clinical data for those subtypes have been reported [9,10].Various treatment modalities are used in HNC, including surgery, chemotherapy, radiotherapy, molecularly-targeted therapy, and immunotherapy [4,5,6,7,8,11,12,13]; the sequence and combination of HNC treatments vary, and treatment policies differ greatly depending on the primary site [14,15,16,17,18,19,20]. It is difficult for the strict eligibility criteria of clinical trials to reflect the diversity of real-world treatment strategies. Therefore, accumulating a large amount of retrospective data from patients with RMHNC who received nivolumab therapy in real-world settings would help validate previous observations [21,22].

The aim of this retrospective multicenter study was to investigate the efficacy of nivolumab in real-world patients with RMHNC and to identify the prognostic factors for response to nivolumab therapy.

## 2. Results

### 2.1. Patient Characteristics

**Figure 1:**
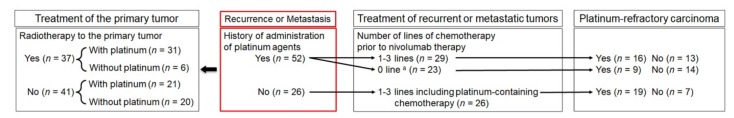
Flow chart of treatment of the primary tumor and recurrent or metastatic tumors. ^a^ Nivolumab was administered as the first systemic therapy for recurrent or metastatic disease.

In total, 93 patients with RMHNC who received nivolumab therapy were included in the study; patient characteristics are summarized in Table 1. Among these patients, 78 had SCC and 15 had non-SCC. The histologies of non-SCC cases included adenoid cystic carcinoma (*n* = 4), salivary duct carcinoma (*n* = 3), lymphoepithelial carcinoma (*n* = 2), adenocarcinoma (*n* = 2), melanoma (*n* = 2), sarcoma (*n* = 1), and synovial sarcoma (*n* = 1). The median follow-up periods for the 93 patients were 36.7 and 23.0 weeks for monitoring of overall survival (OS) and progression-free survival (PFS), respectively. The median follow-up periods for 78 patients with SCC were 38.4 and 24.7 weeks for monitoring of OS and PFS, respectively. The mean age at nivolumab therapy was 64.9 ± 9.9 years, and most patients were male (79.6%), had a smoking history (62.4%), and had a PS of 0 or 1 (92.5%). Primary tumor sites included the pharynx, oral cavity, larynx, salivary glands, nasal cavity and paranasal sinus, thyroid gland, external auditory canal, and unknown sites; the most common primary site was the hypopharynx. The PD-L1 expression level was examined in 22.6% of patients. Radiotherapy to the primary tumor was administered in 46.2% of patients, and surgery and/or chemotherapy were performed in 53.8% of patients. Of the 93 patients, 36 (38.7%), 50 (53.8%), 38 (40.9%), and 19 (20.4%) had confirmed recurrence or metastasis in the primary site, lymph nodes, lung, and other sites, respectively, including the bone, brain, liver, and adrenal gland. Treatment of recurrent or metastatic tumors prior to nivolumab therapy included chemotherapy, cetuximab, surgery, and radiotherapy. Chemotherapy for RMHNC prior to nivolumab therapy was administered in 65 patients (69.9%), of which 55 (59.1%) received platinum-containing chemotherapy, and 28 (30.1%) did not receive chemotherapy or cetuximab; that is, nivolumab was the first systemic therapy for RMHNC given to these 28 patients. The reasons for selecting nivolumab as the first systemic therapy varied and included renal dysfunction, pancytopenia, poor general condition, and poor response to platinum-based induction chemotherapy. Cetuximab administration for the treatment of RMHNC prior to nivolumab therapy was performed in 30 patients (32.3%). Surgery for RMHNC prior to nivolumab therapy including local resection and neck dissection was performed in 24 patients (25.8%), and radiotherapy was administered in 26 patients (28.0%). Tumor progression or recurrence within six months of the last dose of platinum-containing chemotherapy or residual carcinoma after the last platinum-containing regimen was confirmed in 48 patients (51.6%). No history of platinum exposure throughout all the treatments was observed in one malignant melanoma case. At the time of analysis, 22 out of the 93 patients (23.7%) were still receiving nivolumab. The characteristics of the 78 patients with recurrent or metastatic SCC of the head and neck (SCCHN) are also summarized in Table 1, and Figure 1 shows the treatment flow in patients with SCCHN. Among the 78 patients with SCCHN, radiotherapy to the primary tumor was administered in 37 patients, among whom 31 received platinum-containing chemotherapy or chemoradiotherapy. Radiotherapy to the primary tumor was not administered in the remaining 41 patients, among whom 21 received platinum-containing chemotherapy. Consequently, administration of platinum agents for the treatment of the primary tumor was performed in 52 patients with SCCHN, among whom 29 received one to three lines of chemotherapy for recurrent or metastatic tumors prior to nivolumab therapy and 23 received nivolumab as the first systemic therapy for RMHNC. There was no history of administration of platinum agents for the treatment of the primary tumor in 26 patients, who received one to three lines of platinum-containing chemotherapy for recurrent or metastatic tumors prior to nivolumab therapy. If we defined platinum-refractory as having a history of administration of platinum agents within the six months prior to nivolumab use, which was an inclusion criterion for the Checkmate-141 study, 44 (56.4%) patients were platinum-refractory, and 34 (43.6%) patients were not platinum-refractory.

### 2.2. Treatment Efficacy in All Patients Based on Histological Type

The objective response rate (ORR) in all patients was 18.3%; 4.3% and 13.9% of patients achieved complete response (CR) and partial response (PR), respectively. Furthermore, 11.8% of patients had stable disease (SD), and 64.5% had progressive disease (PD); responses were not evaluated in 5.4% of patients (Table 2). The rates of CR, PR, SD, and PD in patients with SCCHN were similar to those in all patients. In contrast, the ORR in patients with non-SCCHN was 0% (Table 2).

**Figure 2:**
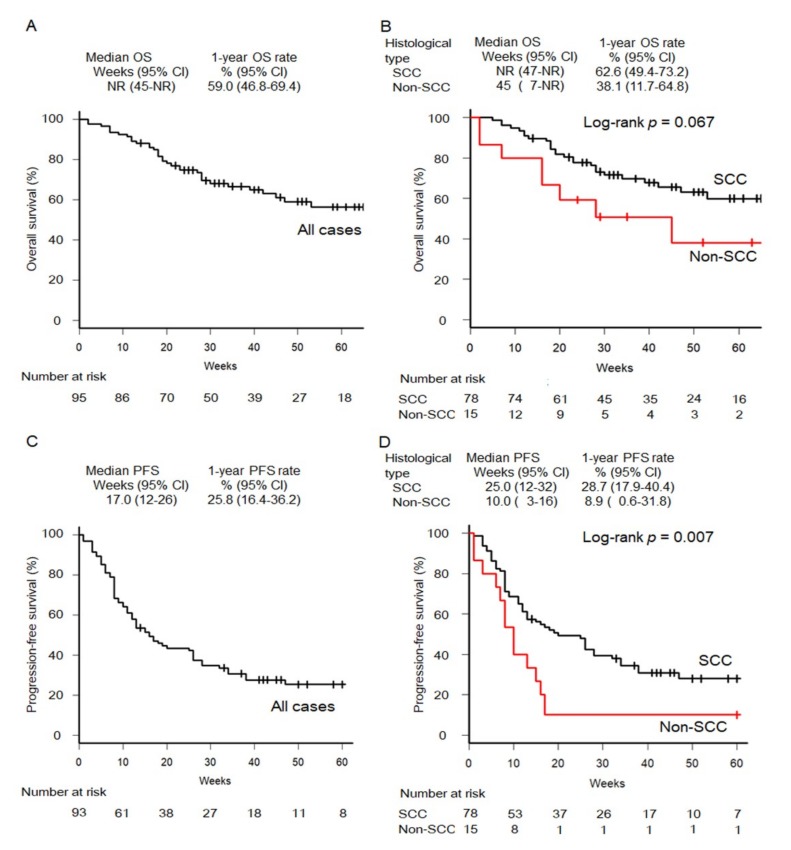
Kaplan-Meier curves of OS in all patients (**A**) and by histological type (**B**). Kaplan-Meier curves of PFS in all patients (**C**) and by histological types (**D**). CI, confidence interval; NR, not reached; OS, overall survival; PFS, progression-free survival; SCC, squamous cell carcinoma.

OS and PFS Kaplan-Meier curves are shown in Figure 2. At the time of analysis, 25 deaths (32.1% of patients) occurred in the SCCHN group, and 8 deaths (53.3%) occurred in the non-SCCHN group; 52 PFS events (66.7% of patients) occurred in the SCCHN group, and 14 events (93.3%) occurred in the non-SCCHN group. Estimated median OS in all patients and in patients with SCCHN was not reached; that in patients with non-SCCHN was 45 weeks. In all patients, the SCCHN group, and the non-SCCHN group, the estimated 1-year OS rates were 59.0%, 62.6%, and 38.1%, respectively. The estimated hazard ratio (HR) for risk of death in the SCCHN group versus the non-SCCHN group was 2.05 (95% confidence interval: 0.93–4.53; log-rank *p* = 0.067). In contrast, the estimated median PFS durations in all patients, patients with SCCHN, and patients with non-SCCHN were 17, 25, and 10 weeks, respectively. In all patients, the SCCHN group, and the non-SCCHN group, the estimated 1-year PFS rates were 25.8%, 28.7%, and 8.9%, respectively. The estimated HR for risk of PFS events for the SCCHN group versus the non-SCCHN group was 2.28 (95% CI: 1.21–4.1; log-rank *p* = 0.007).

### 2.3. Treatment-Related Adverse Events

The frequencies of treatment-related adverse events (TRAEs) that occurred in patients with SCCHN are summarized in Table 3. In 80 patients with SCCHN, 28 TRAEs were detected Baseline characteristics between patients with and without TRAEs were not statistically different. Hypothyroidism (*n* = 5; all grade 2), pneumonia (*n* = 5; grade 1 in 3 cases, grade 2 in 1 case, and grade 3 in 1 case), and skin toxicity (*n* = 5; grade 1 in 1 case and grade 2 in 4 cases) were the most frequent TRAEs, followed by adrenal insufficiency (*n* = 3; all grade 3), colitis/diarrhea (*n* = 3; grade 1 in 2 cases and grade 3 in 1 case), stomatitis (*n* = 2; grade 1 in 1 case and grade 2 in 1 case), liver dysfunction (*n* = 2; grade 1 in 1 case and grade 3 in 1 case), arthritis (*n* = 1, grade 2), diabetes mellitus (*n* = 1, grade 2), and renal dysfunction (*n* = 1, grade 2). Twelve patients who developed TRAEs, including six patients with grade 3 TRAEs, stopped nivolumab treatment; two patients later resumed nivolumab. The median time to onset of TRAEs in all 28 patients was 17.2 weeks (range, 2 to 43 weeks), and those in patients who developed grades 1–2 and 3 events were 14.5 and 27.2 weeks, respectively. The median time to onset of grade 3 TRAEs was significantly longer than that of grade 1–2 TRAEs (*p* = 0.019, Student’s *t*-test).

### 2.4. Association between Prognostic Factors and Treatment Efficacy in Patients with SCCHN

**Figure 3:**
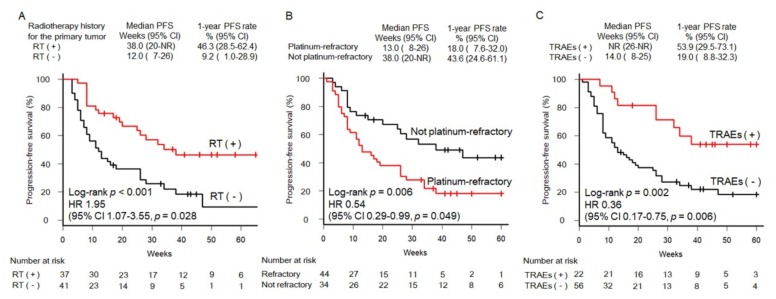
Curves of PFS in patients with SCCHN according to RT history (**A**), platinum-refractory status (**B**), and occurrence of TRAEs (**C**). CI, confidence interval; HR, hazard ratio; NR, not reached; PFS, progression-free survival; RT, radiotherapy; SCCHN, squamous cell carcinoma of the head and neck; TRAEs, treatment-related adverse events.

The results of the univariate analysis of clinical factors affecting 1-year PFS among patients with SCCHN are listed in Table 4. Candidate factors that showed a significant association with PFS included a history of radiotherapy to the primary tumor, platinum-refractory carcinoma, and occurrence of TRAEs. The Kaplan-Meier PFS curves stratified by these factors are shown in Figure 3. The 1-year PFS rate was significantly higher in patients who received radiotherapy to the primary tumor than that in those who received treatments other than radiotherapy (46.3% versus 9.2%, respectively; *p* < 0.001; Figure 3A). The 1-year PFS rate was significantly lower in patients with platinum-refractory carcinoma than in those with non-platinum-refractory carcinoma (18.0% versus 43.6%, respectively; *p* = 0.006; Figure 3B). The 1-year PFS rate was significantly higher in patients who experienced TRAEs than in those who did not experience TRAEs (53.9% versus 19.0%, respectively; *p* = 0.002; Figure 3C). In contrast, the 1-year OS rate was significantly lower in patients with platinum-refractory carcinoma than in those with non-platinum-refractory carcinoma (44.3% versus 84.0%, respectively; *p* = 0.006). However, there were no significant differences in the 1-year OS rate between patients who received radiotherapy to the primary tumor and those who received treatments other than radiotherapy. (*p* = 0.068). In addition, there were no significant differences patients with TRAEs and those without TRAEs (*p* = 0.129).

Multivariate analysis showed that independent prognostic factors included a history of radiotherapy to the primary tumor (HR, 1.95 [95% CI: 1.07–3.55]; *p* = 0.028; Figure 3A) and platinum-refractory carcinoma (HR, 0.54 [95% CI: 0.29–0.99]; *p* = 0.049; Figure 3B). Occurrence of TRAEs was also associated with a longer PFS (HR, 0.36 [95% CI: 0.17–0.75]; *p* = 0.006; Figure 3C). The remaining factors evaluated in this study lost their significance in multivariate analysis.

## 3. Discussion

The current study provided a large amount of efficacy data from a real-world setting. To the best of our knowledge, this is the first retrospective report to investigate the efficacy and prognostic factors of nivolumab therapy in patients with RMHNC in a real-world setting. In real-world settings, we often treat patients with RMHNC who are not eligible for prospective studies, such as Checkmate-141. In the Japanese leaflet for nivolumab, indications for nivolumab include RMHNC; however, the efficacy and safety of nivolumab in patients who have not been treated with platinum-based chemotherapy have not been established. In fact, this study included patients with non-SCC and those with carcinomas of the salivary glands, nasal cavity, paranasal sinus, thyroid gland, external auditory canal, and unknown sites who were not eligible for the Checkmate-141 trial. Thus, the use of nivolumab in patients who did not meet the eligibility criteria for the Checkmate-141 trial was determined by the patient’s doctor based on information from the leaflet.

We identified histology other than SCC as a negative predictor of ORR and PFS. No clinical trials have evaluated nivolumab efficacy for patients with carcinomas of the salivary gland, thyroid gland, nasal cavity, and paranasal sinus, which are usually not SCCs. The current series included two cases of mucosal melanoma of the head and neck. Although recent studies reported that immunotherapy for advanced melanoma, including patients with mucosal melanoma, yielded superior OS, PFS, and ORR compared with chemotherapy, specific data for this subgroup of patients have not been reported [23,24,25]. The prognosis of patients with head and neck mucosal melanoma remains poor. This tumor type has a very high propensity to relapse, regardless of the radicality of resection and adjuvant treatment administered [26]. Although we identified histology other than SCC as a negative predictor in this study, the number of patients with non-SCC was small, making comparisons between SCC and non-SCC unreliable. Therefore, future clinical studies are needed to determine the efficacy of nivolumab treatment in patients with non-SCC.

Univariate and multivariate analyses of factors associated with outcomes in nivolumab-treated patients showed a significant impact of history of radiotherapy to the primary tumor, suggesting that there may be an interaction between nivolumab and irradiation. Irradiation of the tumor can lead to the liberation of tumor cell-derived antigens, which can be recognized and processed by antigen-presenting cells, resulting in priming of circulating cytotoxic T cells. Inhibition of the PD-1/programmed death ligand-1 (PD-L1) signaling pathway can then enhance the efficacy of irradiation through a cytotoxic T cell-dependent mechanism [27]. We further speculated that radiotherapy plus immunotherapy may generate abscopal effects characterized by tumor regression of untreated metastatic lesions after local radiotherapy [28,29,30,31]. Postow et al. [28] reported a case of the abscopal effect in a patient with melanoma treated with ipilimumab and radiotherapy; this patient exhibited increased tumor-antigen production and cytotoxic T cells after radiotherapy in association with tumor shrinkage. Radiotherapy has a much greater chance of generating tumor cell-derived antigens for stimulation of antigen-presenting cells than surgery, which could result in a better immune-checkpoint inhibitor response to recurrent or metastatic tumors. The abscopal effect has been well described in patients with melanoma treated with anti-PD-1 agents and radiotherapy, but evidence for other malignancies, including HNC, is limited [30,31]. In mouse models of SCCHN and melanoma, radiotherapy-induced PD-L1 upregulation was observed in tumors and their microenvironments using immune positron emission tomography/computed tomography [32]. In mouse models injected with mouse oral cancer cells, high-dose hypofractionated radiation preserves or enhances anti-tumor immunity and, when combined with anti-PD-1 monoclonal antibodies to reverse adaptive immune resistance, promotes CD8+ cell-dependent primary and abscopal tumor control [33]. Interestingly, a history of radiotherapy for recurrent or metastatic disease was not associated with a longer PFS, whereas a history of radiotherapy to the primary tumor was associated with longer PFS in the current series. The reason was unclear but may be related to the weaker immunogenicity of recurrent or metastatic disease compared with that of the primary tumor.

We identified platinum-refractory carcinoma as an independent negative predictor of both PFS and OS. Rapid progression after platinum-containing chemotherapy has been shown to be associated with worse outcomes. A global phase III study demonstrated that nivolumab improves the prognosis of patients with platinum-refractory, recurrent, or metastatic SCCHN who experience disease progression within six months after the last platinum-containing regimen [6]; however, in real-world settings, nivolumab is sometimes used as a first-line therapy for RMHNC more than six months after platinum-containing chemotherapy. The current study showed that such patients experienced a better prognosis and a higher probability of response compared with patients treated with nivolumab within six months of the last platinum-containing regimen, which was an eligibility criterion in the Checkmate-141 trial. However, the underlying mechanism for this phenomenon is unknown. Therefore, the use of nivolumab treatment for patients with an interval of greater than six months since the last platinum-containing regimen should be re-evaluated in future clinical trials.

We also found that the development of TRAEs was an independent predictor of a better PFS and was not that of OS. The result for OS may be affected by many survival cases due to the short follow-up period in the current study. Retrospective studies of advanced melanoma and non-small cell lung cancer have shown that PFS and ORR are improved in patients who experienced TRAEs compared with those who did not experience TRAEs [34,35,36,37]. However, similar studies in patients with HNC have not been reported. Recently, a Spanish series of 106 patients with advanced cancer treated with anti-PD-1 agents showed that the patients who developed TRAEs had a significantly better ORR and longer PFS, but not a significantly longer OS [38]. Although only seven patients with SCCHN were included among the 106 total patients in that study, the results of the current series confirmed the findings from the previous study described above, as well as other studies evaluating the association between TRAEs and nivolumab efficacy [34,35,36,37,38].

The limitations of the current study included its retrospective design, short follow-up period, and small population; however, this was the largest multicenter cohort of its kind, thereby providing novel findings. Moreover, the expression levels of PD-L1 and other biomarkers, such as p16, were not consistently examined because diagnostic PD-L1 immunostaining was not covered by Japanese public medical insurance until October 2016. To overcome these limitations, further large-scale studies with long-term follow-up should be performed.

## 4. Materials and Methods

This study was performed in 11 institutes associated with Kyoto University and its Affiliated Hospitals—Head and Neck Oncology Group (Kyoto–HNOG) in Japan. Clinical data for each patient were retrospectively extracted from medical charts and entered into a database. The database included information regarding patient characteristics, treatment modality, histological findings, and clinical outcomes. Patients with RMHNC who received nivolumab monotherapy between May 2017 and May 2018 were eligible for this study. The clinical stages of all patients at diagnosis of primary HNC were classified based on the 8th edition of the TNM classification [39,40]. The Eastern Cooperative Oncology Group (ECOG) performance status (PS) was evaluated just prior to administration of nivolumab. Tumor assessment was performed according to the Response Evaluation Criteria in Solid Tumors (RECIST version 1.1). The ORR corresponded to all cases with a CR or PR. Intervals between the date of commencing nivolumab treatment and that of death alone (OS), disease progression, or death (PFS) were calculated for each patient. To identify prognostic factors, candidate factors were selected, including histology, age greater than 75 years, sex, smoking status, ECOG PS, primary tumor site, PD-L1 expression, a history of radiotherapy to the primary tumor, site of recurrent or metastatic tumors, treatment modality used prior to nivolumab therapy, platinum-refractory carcinoma, and occurrence of TRAEs. PD-L1 expression levels were tested using PDL-1 IHC 28-8 assays. Platinum-refractory carcinoma was defined as a carcinoma that progressed or recurred within 6 months after the last platinum-containing chemotherapy regimen or a residual carcinoma after the last platinum-containing regimen. To analyze safety, severe toxicities related to nivolumab therapy (grade ≥ 1 according to the Common Terminology Criteria for Adverse Events version 4.0) were investigated. The data cutoff point for the analyses was 30 September 2018, which was the date of the planned interim analysis. This study was approved by the institutional review board of each participating institution and was led by the Kyoto University Certificated Review Board (ethics code: R1469). Informed consent was not required owing to the retrospective nature of this study.

For statistical analysis, clinical outcomes, including PFS and OS, were estimated using the Kaplan-Meier method, and groups were compared using the log-rank test. Cox proportional hazards regression models were used to determine associations between patient characteristics and PFS outcomes. Multivariate Cox proportional hazards regression was performed for each variable identified as statistically significant by univariate analysis. Results with *p* values of less than 0.05 were regarded as statistically significant. SPSS software (version 22.0; IBM Corp., Armonk, NY, USA) was used for statistical analysis.

## 5. Conclusions

In this report, we demonstrated the efficacy of nivolumab in a real-world setting. The current study provided a large amount of efficacy data in patient types that are relatively less common and thus under-represented in clinical trials. The results of this study suggested that the efficacy of nivolumab in patients with non-SCC was inferior to that in patients with SCC and revealed that a history of radiotherapy to the primary tumor, platinum-refractory carcinoma, and development of TRAEs were important prognostic factors associated with PFS in patients with nivolumab-treated SCCHN. These findings could be useful for clinicians and patients when determining treatment strategy. Considering the limitations of our cohort, future prospective studies are needed to confirm the current findings.

## Figures and Tables

**Table 1:** Patient characteristics.

Variable			All Patients (*N* = 93)	SCCHN Patients ^a^ (*N* = 78)
Age				
	75 or higher, *n* (%)		15 (16.1)	11 (14.1)
	<75, *n* (%)		78 (83.9)	67 (85.9)
	Mean ± SD (years)		64.9 ± 9.9	64.9 ± 9.7
Sex				
	Male, *n* (%)		74 (79.6)	65 (83.3)
	Female, *n* (%)		19 (20.4)	13 (16.7)
Smoking status, *n* (%)				
	(Current or former) Smoker		58 (62.4)	49 (62.8)
	Never-smoker		35 (37.6)	29 (37.1)
ECOG PS, *n* (%)				
	0 or 1		86 (92.5)	71 (91.0)
	2 or 3 or 4		7 (7.5)	7 (9.0)
Primary tumor site, *n* (%)				
	Pharynx		45 (48.4)	39 (50.0)
		6 (6.5)	4 (5.1)	4 (5.0)
		11 (11.8)	11 (14.1)	12 (15.0)
		26 (28.0)	24 (30.8)	25 (31.3)
	Oral cavity		15 (16.1)	14 (18.0)
	Larynx		6 (6.5)	6 (7.7)
	Salivary gland		6 (6.5)	-
		Parotid gland	3 (3.2)	-
		Submandibular gland	2 (2.2)	-
		Sublingual gland	1 (1.1)	-
	Nasal cavity and paranasal sinus		10 (10.8)	8 (12.8)
	Thyroid gland		2 (2.2)	1 (1.3)
	External auditory canal		3 (3.2)	3 (3.9)
	Unknown primary		8 (8.6)	7 (9.0)
PD-L1 expression				
	<1%		5 (5.4)	5 (6.4)
	1–10%		6 (6.5)	6 (7.7)
	≥10%		10 (10.8)	10 (12.8)
	Not evaluated		74 (77.9)	72 (77.4)
Radiotherapy to the primary tumor, *n* (%)				
	Radiotherapy (+)		43 (46.2)	37 (47.4)
	Radiotherapy (−)		50 (53.8)	41 (52.6)
History of administration of platinum agents for the treatment of primary tumor, *n* (%)				
	Platinum agents (+)		60 (64.5)	52 (66.7)
	Platinum agents (−)		33 (45.5)	26 (33.3)
Site of recurrent or metastatic tumors, *n* (%)				
	Local primary site		36 (38.7)	33 (42.3)
	Lymph nodes		50 (53.8)	44 (56.4)
	Lung		38 (40.9)	27 (34.6)
	Others		19 (20.4)	9 (11.8)
Treatment of recurrent or metastatic tumors prior to nivolumab therapy, *n* (%)				
	Chemotherapy		65 (69.9)	55 (70.5)
		Number of lines		
		1 line	32 (34.4)	29 (37.2)
		2 lines	24 (25.8)	20 (25.0)
		3 lines	9 (9.7)	6 (7.5)
		0 line ^b^	28 (30.1)	23 (29.5)
	Platinum-containing chemotherapy		55 (57.9)	55 (59.1)
	Chemotherapy plus cetuximab		30 (31.6)	30 (32.3)
	Surgery		24 (25.3)	24 (25.8)
		Local resection	11 (11.8)	11 (14.1)
		Neck dissection	17 (18.3)	16 (20.5)
	Radiotherapy		27 (28.4)	26 (28.0)
Platinum-refractory carcinoma ^c^, *n* (%)				
	Platinum-refractory		48 (51.6)	44 (56.4)
	Not platinum-refractory		44 (47.3)	34 (43.6)
	No history of a platinum-containing regimen ^d^		1 (1.1)	

^a^ Patients with SCCHN. ^b^ Nivolumab was administered as the first systemic therapy for recurrent or metastatic disease. ^c^ A carcinoma that progressed within 6 months after the last platinum-containing regimen or a residual carcinoma after the last platinum-containing regimen. ^d^ No history of platinum exposure was observed in one malignant melanoma case. ECOG, Eastern Cooperative Oncology Group; PD-L1, programmed death ligand-1; PS, performance status; SCCHN, squamous cell carcinoma of the head and neck; SD, standard deviation.

**Table 2:** Best overall response in patients.

Best Overall Response	All Patients (*N* = 93)	SCCHN Patients ^a^ (*N* = 78)	Non-SCCHN Patients ^b^ (*N* = 15)
Complete Response, *n* (%)	4 (4.3)	4 (5.1)	0 (0.0)
Partial Response, *n* (%)	13 (13.9)	13 (16.7)	0 (0.0)
Stable Disease, *n* (%)	11 (11.8)	10 (12.8)	1 (6.7)
Progressive Disease, *n* (%)	60 (64.5)	49 (62.8)	11 (73.3)
Not Evaluable or Assessed, *n* (%)	5 (5.4)	2 (2.6)	3 (20.0)
ORR (CR + PR), *n* (%)	17 (18.3)	17 (21.8)	0 (0.0)

^a^ Patients with SCCHN. ^b^ Patients with non-SCCHN. CR, complete response; ORR, objective response rate; PR, partial response; SCCHN, squamous cell carcinoma of the head and neck.

**Table 3:** Treatment-related adverse events in patients with SCCHN.

TRAEs	*n* (%)	Grade 1 *n*	Grade 2 *n*	Grade 3 *n*
Overall	28 (35.9)	8	14	6
Hypothyroidism	5 (6.4)		5	
Pneumonia	5 (6.4)	3	1	1
Skin Toxicity	5 (6.4)	1	4	
Adrenal Insufficiency	3 (3.8)			3
Colitis/Diarrhea	3 (3.8)	2		1
Stomatitis	2 (2.6)	1	1	
Liver Dysfunction	2 (2.6)	1		1
Arthritis	1 (1.3)		1	
Diabetes Mellitus	1 (1.3)		1	
Renal Dysfunction	1 (1.3)		1	

SCCHN, squamous cell carcinoma of the head and neck; TRAEs, treatment-related adverse events.

**Table 4:** Univariate analysis of clinical factors associated with the 1-year PFS rate among patients with SCCHN.

Variable			PFS Rate (1 Year)	(95% CI)	*p*
Age (years)					0.406
		75 or higher (*n* = 11)	39.8%	(11.0–68.0)	
		<75 (*n* = 67)	27.1%	(15.9–39.4)	
Sex					0.618
		Male (*n* = 65)	29.5%	(17.0–43.2)	
		Female (*n* = 13)	23.1%	(5.6–47.5)	
Smoking statuse					0.574
		Smoker (*n* = 49)	33.1%	(19.2–47.7)	
		Never-smoker (*n* = 29)	25.5%	(11.1–42.6)	
ECOG PS					0.257
		0 or 1 (*n* = 71)	31.4%	(19.8–43.8)	
		2 or 3 or 4 (*n* = 7)	Not reached		
PD-L1 expression					0.82
		<1% (*n* = 5)	26.7%	(1.0–68.6)	
		≥1% (*n* = 16)	24.6%	(4.6–52.8)	
Radiotherapy to the primary tumor					< 0.001 *
		Radiotherapy (+) (*n* = 37)	46.3%	(28.5–62.4)	
		Radiotherapy (−) (*n* = 41)	9.2%	(1.0–28.9)	
Site of recurrernt or metastatic tumors					
	Local primary site				0.654
		Yes (*n* = 33)	27.1%	(10.9–46.4)	
		No (*n* = 45)	30.2%	(17.1–44.5)	
	Lymph nodes				0.262
		Yes (*n* = 44)	25.5%	(12.8–40.3)	
		No (*n* = 34)	33.2%	(15.9–51.6)	
	Lung				0.061
		Yes (*n* = 27)	19.3%	(6.7–36.6)	
		No (*n* = 51)	33.6%	(18.9–48.9)	
Treatment of recurrent or metastatic tumors prior to nivolumab therapy					
	Chemotherapy				0.899
		Yes (*n* = 55)	32.0%	(19.7–44.9)	
		No (*n* = 23)	14.8%	(1.3–43.4)	
	Platinum-containing chemotherapy				0.598
		Yes (*n* = 48)	30.0%	(17.2–44.0)	
		No (*n* = 30)	25.8%	(8.9–46.9)	
	Chemotherapy plus cetuximab				0.071
		Yes (*n* = 27)	23.3%	(9.1–41.2)	
		No (*n* = 51)	32.6%	(18.8–47.1)	
	Surgery				0.419
		Yes (*n* = 22)	24.5%	(8.7–44.6)	
		No (*n* = 56)	30.4%	(17.1–44.8)	
	Radiotherapy				0.154
		Yes (*n* = 22)	25.5%	(9.3–45.4)	
		No (*n* = 56)	30.3%	(17.2–44.3)	
Platinum-refractory carcinoma ^a^					0.006 *
		Platinum-refractory (*n* = 44)	18.4%	(7.6–32.0)	
		Not platinum-refractory (*n* = 34)	43.6%	(24.6–61.1)	
Occurrence of TRAEs					0.002 *
		TRAEs (+) (*n* = 22)	53.9%	(29.5–73.1)	
		TRAEs (−) (*n* = 56)	19.0%	(8.8–32.3)	

^a^ A carcinoma that progressed within six months after the last platinum-containing regimen or a residual carcinoma after the last platinum-containing regimen. CI, confidence interval; ECOG, Eastern cooperative oncology group; PFS, progression-free survival rate; SCCHN, squamous cell carcinoma of the head and neck; TRAEs, treatment-related adverse events. * Statistically significant.

## References

[1] Ferlay J., Soerjomataram I., Dikshit R., Eser S., Mathers C., Rebelo M., Parkin D.M., Forman D., Bray F. (2015). Cancer incidence and mortality worldwide: Sources, methods and major patterns in GLOBOCAN 2012. Int. J. Cancer..

[2] Hori M., Matsuda T., Shibata A., Katanoda K., Sobue T., Nishimoto H., Japan Cancer Surveillance Research Group (2015). Cancer incidence and incidence rates in Japan in 2009: A study of 32 population-based cancer registries for the Monitoring of Cancer Incidence in Japan (MCIJ) project. Jpn. J. Clin. Oncol..

[3] Pignon J.P., le Maître A., Maillard E., Bourhis J. (2009). Meta-analysis of chemotherapy in head and neck cancer (MACH-NC): An update on 93 randomised trials and 17,346 patients. Radiother. Oncol..

[4] Bernier J., Domenge C., Ozsahin M., Matuszewska K., Lefèbvre J.L., Greiner R.H., Giralt J., Maingon P., Rolland F., Bolla M. (2004). Postoperative irradiation with or without concomitant chemotherapy for locally advanced head and neck cancer. N. Engl. J. Med..

[5] Cooper J.S., Pajak T.F., Forastiere A.A., Jacobs J., Campbell B.H., Saxman S.B., Kish J.A., Kim H.E., Cmelak A.J., Rotman M. (2004). Postoperative concurrent radiotherapy and chemotherapy for high-risk squamous cell carcinoma of the head and neck. N. Engl. J. Med..

[6] Ferris R.L., Blumenschein G. Jr., Fayette J., Guigay J., Colevas A.D., Licitra L., Harrington K., Kasper S., Vokes E.E., Even C. (2016). Nivolumab for Recurrent Squamous-Cell Carcinoma of the Head and Neck. N. Engl. J. Med..

[7] Vermorken J.B., Mesia R., Rivera F., Remenar E., Kawecki A., Rottey S., Erfan J., Zabolotnyy D., Kienzer H.R., Cupissol D. (2008). Platinum-based chemotherapy plus cetuximab in head and neck cancer. N. Engl. J. Med..

[8] Bonner J.A., Harari P.M., Giralt J., Azarnia N., Shin D.M., Cohen R.B., Jones C.U., Sur R., Raben D., Jassem J. (2006). Radiotherapy plus cetuximab for squamous-cell carcinoma of the head and neck. N. Engl. J. Med..

[9] Takemoto K., Miyahara N., Chikuie N., Hamamoto T., Ishino T., Ueda T., Takeno S. (2018). Efficacy of anti-PD-1 therapy in a patient with brain metastasis of parotid carcinoma: A case report. Auris Nasus Larynx.

[10] Cabezas-Camarero S., Puebla F., Subhi-Issa A.I., Sanz-Ortega J., Pérez-Segura P. (2018). Durable response to first-line nivolumab in a patient with oligometastatic PD-L1 positive nasopharyngeal cancer. Oral Oncol..

[11] Nibu K., Hayashi R., Asakage T., Ojiri H., Kimata Y., Kodaira T., Nagao T., Nakashima T., Fujii T., Fujii H. (2017). Japanese Clinical Practice Guideline for Head and Neck Cancer. Auris Nasus Larynx.

[12] Gooi Z., Fakhry C., Goldenberg D., Richmon J., Kiess A.P. (2016). AHNS Series: Do you know your guidelines? Principles of radiation therapy for head and neck cancer: A review of the National Comprehensive Cancer Network guidelines. Head Neck.

[13] Fulcher C.D., Haigentz M. Jr., Ow T.J. (2018). Education Committee of the American Head and Neck Society (AHNS). AHNS Series: Do you know your guidelines? Principles of treatment for locally advanced or unresectable head and neck squamous cell carcinoma. Head Neck.

[14] Gill A., Vasan N., Givi B., Joshi A. (2018). AHNS Series: Do you know your guidelines? Evidence-based management of oral cavity cancers. Head Neck.

[15] Byrd J.K., Clair J.M., El-Sayed I. (2018). AHNS Series: Do you know your guidelines? Principles for treatment of cancer of the paranasal sinuses: A review of the National Comprehensive Cancer Network guidelines. Head Neck.

[16] Gooi Z., Richmon J., Agrawal N., Blair E., Portugal L., Vokes E., Seiwert T., de Souza J., Saloura V., Haraf D. (2017). AHNS Series - Do you know your guidelines? Principles of treatment for nasopharyngeal cancer: A review of the National Comprehensive Cancer Network guidelines. Head Neck.

[17] Guo T., Goldenberg D., Fakhry C. (2017). AHNS series: Do you know your guidelines? Management of head and neck cancer in the era of human papillomavirus: Educating our patients on human papillomavirus. Head Neck.

[18] Tamaki A., Miles B.A., Lango M., Kowalski L., Zender C.A. (2018). AHNS series: Do you know our guidelines? Review of current knowledge on laryngeal cancer. Head Neck.

[19] Mantravadi A.V., Moore M.G., Rassekh C.H. (2019). AHNS series: Do you know your guidelines? Diagnosis and management of salivary gland tumors. Head Neck.

[20] Eskander A., Ghanem T., Agrawal A. (2018). Education Committee of American Head and Neck Society (AHNS). AHNS Series: Do you know your guidelines? Guideline recommendations for head and neck cancer of unknown primary site. Head Neck.

[21] Donia M., Ellebaek E., Øllegaard T., Duval L., Aaby J., Hoejberg L., Køhler U.H., Schmidt H., Bastholt L., Svane I.M. (2018). The real-world impact of modern treatments on the survival of patients with metastatic melanoma. Eur. J. Cancer.

[22] Al-Baimani K., Jonker H., Zhang T., Goss G.D., Laurie S.A., Nicholas G., Wheatley-Price P. (2018). Are clinical trial eligibility criteria an accurate reflection of a real-world population of advanced non-small-cell lung cancer patients?. Curr. Oncol..

[23] Weber J.S., D’Angelo S.P., Minor D., Hodi F.S., Gutzmer R., Neyns B., Hoeller C., Khushalani N., Miller W.H., Lao C.D. (2015). Nivolumab versus chemotherapy in patients with advanced melanoma who progressed after anti-CTLA-4 treatment (CheckMate 037): A randomised, controlled, open-label, phase 3 trial. Lancet Oncol..

[24] Larkin J., Chiarion-Sileni V., Gonzalez R., Grob J.J., Cowey C.L., Lao C.D., Schadendorf D., Dummer R., Smylie M., Rutkowski P. (2015). Combined Nivolumab and Ipilimumab or Monotherapy in Untreated Melanoma. N. Engl. J. Med..

[25] Wolchok J.D., Chiarion-Sileni V., Gonzalez R., Rutkowski P., Grob J.J., Cowey C.L., Lao C.D., Wagstaff J., Schadendorf D., Ferrucci P.F. (2017). Overall Survival with Combined Nivolumab and Ipilimumab in Advanced Melanoma. N. Engl. J. Med..

[26] Ascierto P.A., Accorona R., Botti G., Farina D., Fossati P., Gatta G., Gogas H., Lombardi D., Maroldi R., Nicolai P. (2017). Mucosal melanoma of the head and neck. Crit. Rev. Oncol. Hematol..

[27] Deng L., Liang H., Burnette B., Beckett M., Darga T., Weichselbaum R.R., Fu X.Y. (2014). Irradiation and anti-PD-L1 treatment synergistically promote antitumor immunity in mice. J. Clin. Invest..

[28] Postow M.A., Callahan M.K., Barker C.A., Yamada Y., Yuan J., Kitano S., Mu Z., Rasalan T., Adamow M., Ritter E. (2012). Immunologic correlates of the abscopal effect in a patient with melanoma. N. Engl. J. Med..

[29] Chandra R.A., Wilhite T.J., Balboni T.A., Alexander B.M., Spektor A., Ott P.A., Ng A.K., Hodi F.S., Schoenfeld J.D. (2015). A systematic evaluation of abscopal responses following radiotherapy in patients with metastatic melanoma treated with ipilimumab. Oncoimmunology.

[30] Grimaldi A.M., Simeone E., Giannarelli D., Muto P., Falivene S., Borzillo V., Giugliano F.M., Sandomenico F., Petrillo A., Curvietto M. (2014). Abscopal effects of radiotherapy on advanced melanoma patients who progressed after ipilimumab immunotherapy. Oncoimmunology.

[31] Ribeiro Gomes J., Schmerling R.A., Haddad C.K., Racy D.J., Ferrigno R., Gil E., Zanuncio P., Buzaid A.C. (2016). Analysis of the Abscopal Effect With Anti-PD1 Therapy in Patients With Metastatic Solid Tumors. J. Immunother..

[32] Kikuchi M., Clump D.A., Srivastava R.M., Sun L., Zeng D., Diaz-Perez J.A., Anderson C.J., Edwards W., Ferris R.L. (2017). Preclinical immunoPET/CT imaging using Zr-89-labeled anti-PD-L1 monoclonal antibody for assessing radiation-induced PD-L1 upregulation in head and neck cancer and melanoma. Oncoimmunology.

[33] Morisada M., Clavijo P.E., Moore E., Sun L., Chamberlin M., Van Waes C., Hodge J.W., Mitchell J.B., Friedman J., Allen C.T. (2017). PD-1 blockade reverses adaptive immune resistance induced by high-dose hypofractionated but not low-dose daily fractionated radiation. Oncoimmunology.

[34] Sato K., Akamatsu H., Murakami E., Sasaki S., Kanai K., Hayata A., Tokudome N., Akamatsu K., Koh Y., Ueda H. (2018). Correlation between immune-related adverse events and efficacy in non-small cell lung cancer treated with nivolumab. Lung Cancer.

[35] Haratani K., Hayashi H., Chiba Y., Kudo K., Yonesaka K., Kato R., Kaneda H., Hasegawa Y., Tanaka K., Takeda M. (2018). Association of immune-related adverse events with nivolumab efficacy in non-small-cell lung cancer. JAMA Oncol..

[36] Weber J.S., Hodi F.S., Wolchok J.D., Topalian S.L., Schadendorf D., Larkin J., Sznol M., Long G.V., Li H., Waxman I.M. (2017). Safety Profile of Nivolumab Monotherapy: A Pooled Analysis of Patients With Advanced Melanoma. J. Clin. Oncol..

[37] Judd J., Zibelman M., Handorf E., O’Neill J., Ramamurthy C., Bentota S., Doyle J., Uzzo R., Bauman J., Borghaei H. (2017). Immune-Related Adverse Events as a Biomarker in Non-Melanoma Patients Treated with Programmed Cell Death 1 Inhibitors. Oncologist.

[38] Rogado J., Sánchez-Torres J.M., Romero-Laorden N., Ballesteros A.I., Pacheco-Barcia V., Ramos-Leví A. (2019). Immune-related adverse events predict the therapeutic efficacy of anti-PD-1 antibodies in cancer patients. Eur. J. Cancer.

[39] James D.B., Mary K.G., Christian W. (2017). TNM Classification of Malignant Tumors.

[40] Amin M.B., Edge S.B., Greene F.L., Byrd D.R., Brookland R.K., Washington M.K., Gershenwald J.E., Compton C.C., Hess K.R., Sullivan D.C. (2017). American Joint Committee on Cancer (AJCC) Cancer Staging Manual.

